# Clinicopathological Predisposing Factors for Gastric Stump Cancer and Its Management: A Single-Center Analytical Study

**DOI:** 10.7759/cureus.44798

**Published:** 2023-09-06

**Authors:** Prashanth Sangu, Sharath Kumar V, Rajkumar Rathinasamy, Prabhakaran R, Sugumar Chidambaranathan, Naganath Babu O L

**Affiliations:** 1 Surgical Gastroenterology, Madras Medical College, Chennai, IND

**Keywords:** curative surgery, clinicopathological factors, gastric stump canrcinoma, gastrojejunostomy, truncal vagotomy

## Abstract

Background

The incidence of gastric stump carcinoma (GSC) is not declining because of the long latency period. The survival rate of treated gastric cancer patients has increased due to early detection and improvements in surgical techniques and chemotherapy. Increased survival rates and improved surveillance following gastric surgery have increased the incidence of GSC.

Aim

The study aims to investigate the clinicopathological factors affecting the interval between index gastric surgery and the occurrence of GSC, and our experience in the management of GSC is presented.

Methods

A retrospective review of patients diagnosed with GSC in our institution was completed. Patient characteristics and clinicopathological outcomes were analyzed.

Results

A total of 28 patients were included in this cohort with 17 (60.71%) males and 11 (39.28%) females. The mean interval from index surgery to the incidence of GSC was 24.42 years for benign etiology and six years for malignant etiology. Index surgeries were truncal vagotomy with 14 gastrojejunostomies (50%) and 14 subtotal gastrectomies (50%). The interval between index surgery and the incidence of GSC is not statistically significant concerning the type of surgery (p: 0.661), pathological TNM (tumor, nodes, metastases) stage (p: 0.520), pathological differentiation (p: 0.828), lymphovascular invasion (p: 0.252), perineural invasion (p: 0.672), and adjuvant therapy (p: -0.655). Survival was significantly higher in those patients who received curative resection in comparison to a palliative procedure (p: 0.041).

Conclusion

Strict surveillance for at least 10 years after initial gastric surgery is of utmost importance as half of the patients fated to develop GSC will do so within this time. In those patients with early diagnosis, no evidence of metastasis, and good performance status, curative surgery is feasible with acceptable morbidity.

## Introduction

Gastric stump cancer (GSC) is the development of malignant tumors associated with previous gastric surgery. Balfour first described it as gastric cancer that develops in the residual stomach after partial gastrectomy for benign or malignant disease [1]. The GSC incidence rates, which are reported to be in the range of 1-9%, do not decrease linearly due to the long incubation period required for carcinogenesis [2,3]. The prevalence of GSC is estimated to be 2.2-3.0% due to its relative rarity [4]. The characteristics of GSC are not fully understood. As surgical treatment for benign disease continues to decline, the number of patients with GSC who have undergone surgical treatment for benign disease continues to decline. Conversely, improved outcomes in patients with gastric cancer have led to an increase in the number of GSC patients after gastrectomy for gastric cancer [5].

GSC occurs earlier after gastrectomy for malignant disease than for benign disease, and patients with GSC have a significantly worse prognosis than patients with primary gastric cancer [6]. GSC is a more advanced gastric cancer that is usually associated with a relatively low curative resection rate [7] because there are no typical signs or symptoms in the early stages. However, the clinicopathological features and prognosis of GSC compared to primary gastric cancer remain controversial. Surgical treatment for patients with GSC is difficult. It is affected by the advanced nature of the disease, changes in anatomical structure from previous surgery, abnormal lymphatic formation from previous surgery, and the patient's immunological status, especially post-surgery GSC for malignant etiology. Therefore, proper patient selection is paramount to achieving curative resection.

Identifying the factors that influence the worse outcomes of GSC is paramount to enabling early diagnosis and curative treatment. This study presents our experience in the management of GSC patients who have undergone surgery for either benign or malignant etiology and factors affecting the interval between index gastric surgery and the onset of GSC. Risk factors, short-term outcomes, and mortality were also analyzed.

## Materials and methods

This is a retrospective analysis of the database prospectively maintained at Madras Medical College, Chennai, India, between 2015 and 2020. Included patients were diagnosed with surgically treated GSC. The study was approved by the Institutional Ethical Committee of Madras Medical College (approval number: 020148). The diagnosis of GSC requires that the interval between primary gastric surgery and the onset of GSC is greater than five years. Previous indications for gastric surgery can be either benign or malignant. A total of 28 patients were included. Medical records were reviewed for the following information: demographic factors, comorbidities, performance status, diagnosis and stage of primary disease, surgical methods of primary disease, intervals between primary and secondary surgery, follow-up method, GSC characteristics (symptoms, location, histology, macroscopic type, and stage), surgical procedure for the second surgery (curative resection rate, combined resection, duration, and hospitalization), and follow-up data.

We adopted the definition of GSC from the Japanese Classifications and Treatment Guidelines for Gastric Cancer (14th edition), in which GSC is defined as a carcinoma arising in the gastric remnant after gastrectomy, regardless of the histology of the previous lesion (benign or malignant), the risk of recurrence, the extent of initial resection, or methods of reconstruction [8]. For preoperative evaluation, all patients underwent upper gastrointestinal endoscopy, chest x-ray, abdominal ultrasonography, or abdominal computer tomography for clinical tumor staging. Positron emission tomography (PET)-CT was performed for resectable lesions only when conventional diagnostic imaging detects suspicious metastatic lesions. Elective surgery was performed on 27 patients and emergency surgery was performed on one patient. Diagnostic laparoscopy was performed in an open technique for all patients who have undergone elective surgery. The carcinomas were staged according to the American Joint Committee on Cancer TNM Staging of Gastric Cancer, 8th edition [9]. The main location of each GSC was categorized as being at the anastomotic or non-anastomotic site and as having diffuse involvement of the remnant stomach. Histology was classified as well-differentiated, moderately differentiated, or poorly differentiated adenocarcinoma. Surgical mortality was defined as occurring within the first 30 days after surgery or during postoperative hospitalization. 

Follow-up included regular visits for history and clinical examinations, chest x-ray, upper gastrointestinal endoscopy (UGIE), and ultrasonography or CT for the first five years (every three months for the initial two years followed by every six months up to five years) and every year thereafter. 

Statistical analysis

Statistical Analysis was performed using IBM SPSS Statistics for Windows, Version 21.0 (released 2012; IBM Corp., Armonk, New York, United States). The association between mortality, curative resection, and palliative resection was assessed by cross-tabulation and comparison of percentages. Chi-square test was used to test statistical significance. The association between primary procedure, grade of differentiation, pathological stage, adjuvant therapy, and interval (in years: 5, 6, 7, and 8) was assessed by cross-tabulation and comparison of percentages. Chi-square test was used to test statistical significance. A univariate binary logistic regression analysis was performed to test the association between post-surgical clinicopathological factors of GSC and short-term outcomes. The unadjusted OR along with 95% CI is presented. p-value < 0.05 was considered statistically significant.

## Results

A total of 28 patients were analyzed and their characteristics are listed in Table 1. The male-female ratio was 1.54 to 1 and the median age was 52.5 +/- 11.7. Of the 28 patients, 14 (50%) had surgery for benign disease (sequelae of gastric ulcer and duodenal ulcer) and 14 (50%) had surgery for gastric cancer. The background of patients from previous surgery is also listed in Table 1. Patients underwent truncal vagotomy with gastrojejunostomy (50%) and D2 subtotal gastrectomy (50%) as the primary procedure. All the primary surgeries done for malignant etiologies were radical resection (R0 resection). Comparing patients with a history of benign disease with those with a history of malignant tumors, there was a significant difference in the time interval between the first and second surgeries (P <0.001).

**Table 1:** Demographic parameters of patients with GSC GSC: gastric stump carcinoma; TV/GJ: truncal vagotomy/gastrojejunostomy; JJ/FJ: jenunojejunostomy/feeding jejunostomy; WDAC: well-differentiated adenocarcinoma; MDAC: moderately differentiated adenocarcinoma; PDAC: poorly differentiated adenocarcinoma

Demographics	Total (n=28)	Benign (n=14)	Malignant (n =14)
Age (years), mean ± SD	52.50 ± 11.76	55.71 ± 10.54	47.35 ± 12.41
Sex, n (%)			
Male	17 (60.71%)	11 (78.57%)	6 (42.85%)
Female	11 (39.28%)	3 (21.42%)	8 (57.15%)
Primary procedure, n (%)			
TV/GJ	14 (50%)	14 (100%)	0 (0)
Subtotal gastrectomy	14 (50%)	0 (0%)	14 (100%)
Time interval for the occurrence of GSC (years), mean ± SD	15.24 ± 11.46	24.42 ± 9.44	6 ± 0.96
5 -10 years, n )%)	14 (50%)	0 (0%)	14 (100%)
10-20 years, n (%)	5 (17.85%)	5 (35.71%)	0 (0%)
>20 years, n (%)	9 (32.14%)	9 (64.28%)	0 (0%)
Site of recurrence, n (%)			
Anastomotic	19 (67.85%)	9 (64.28%)	10 (71.42%)
Non-anastomotic	7 (25%)	3 (21.42%)	4 (28.56%)
Diffuse	2 (7.14%)	2 (14.28%)	0 (0%)
Borrmann type (GSC), n (%)			
1	21 (75%)	10 (71.42%)	11 (78.57%)
2	5 (17.85%)	3 (21.42%)	2 (14.28%)
3	1 (3.57%)	0 (0%)	1 (7.14%)
4	0 (0%)	0 (0%)	0 (0%)
5	1 (3.57%)	1 (7.14%)	0 (0%)
Type of procedure performed for GSC, n (%)			
Feeding jejunostomy	14 (50%)	9 (64.28%)	5 (35.71%)
JJ/FJ	4 (14.28%)	2 (14.28%)	2 (14.28%)
Total gastrectomy	3 (10.71%)	3 (21.42%)	0 (0%)
Completion gastrectomy	2 (7.14%)	0 (0%)	2 (14.28%)
Resection of recurrence	4 (14.28%)	0 (0%)	4 (28.57%)
PTBD	1 (3.57%)	0 (0%)	1 (7.14%)
Pathological stage (after surgery for GSC), n (%)			
Stage 1	0 (0%)	0 (0%)	0 (0%)
Stage 2	3 (10.71%)	0 (0%)	3 (21.42%)
Stage 3	4 (14.28%)	2 (14.28%)	2 (14.28%)
Stage 4	21 (75%)	12 (85.71%)	9 (64.28%)
Pathological differentiation after surgery for GSC, n (%)			
WDAC	1 (3.57%)	1 (7.14%)	0 (0%)
MDAC	9 (32.14%)	5 (35.7%)	4 (28.6%)
PDAC	10 (35.71%)	5 (35.7%)	5 (35.71%)
Diagnosed with metastatic deposits (e.g., ascites analysis, omental, peritoneal)	8 (28.57%)	3 (21.4%)	5 (35.71%)
Follow-up, n (%)			
Expired	17 (60.7%)	8 (57.14%)	9 (64.28%)
Alive	11 (39.3%)	6 (42.85%)	5 (35.71%)

In patients with primary malignancies, early stages I, II, and III according to the 8th edition of the TNM classification were 7.14%, 28.57%, and 64.28%, respectively. Histological differentiation was poor in the majority of patients (n=8; 57.14%), with well and moderate differentiation observed in one (7.14%) and five (35.71%) patients, respectively. Lymphovascular invasion and perineural infiltration were observed in 11 (78.57%) and four (28.57%), respectively.

The multidisciplinary team decided on an adjuvant chemotherapy regimen. Of the 14 patients with primary malignancies, 11 (39.28%) completed the proposed chemotherapy and two (7.14%) discontinued treatment. One patient did not received any adjuvant chemotherapy.

All patients exhibited one or more symptoms of abdominal pain, vomiting, dysphagia, bloating, jaundice, weight loss, and loss of appetite. Recurrences occurred in 19 (67.85%) patients at the previous anastomotic site, and in seven (25%) patients at the non-anastomotic sites such as lymph nodes, proximal to the anastomosis of gastric remnant, and diffuse pattern involving the entire stomach noted in the remaining two patients (7.14%). Distant metastases were reported in areas such as liver (n=6; 21.42%), omentum (n=7; 25%), and peritoneum (n=8; 28.57%). Involvement of adjacent organs such as the pancreas, small intestine (jejunum), and transverse colon was observed in four patients (14.28%) with GSC.

The interval between the first and second surgeries in the patient population varied significantly depending on the underlying disorder (p <0.001). Patients with a history of benign disease developed GSC median 30 years after previous surgery (mean 24.42 ± 9.44 years). On the other hand, all patients with a history of gastric cancer developed GSC by 5-10 years of primary surgery (mean 6.0 ± 0.96). The interval between index surgery and GSC incidence is not statistically significant with respect to the type of surgery (p: 0.409), pathological TNM stage (p: 0.574), pathological differentiation and vascular invasion (p: 0.747), and perineural invasion (p: 0.6).

**Figure 1:**
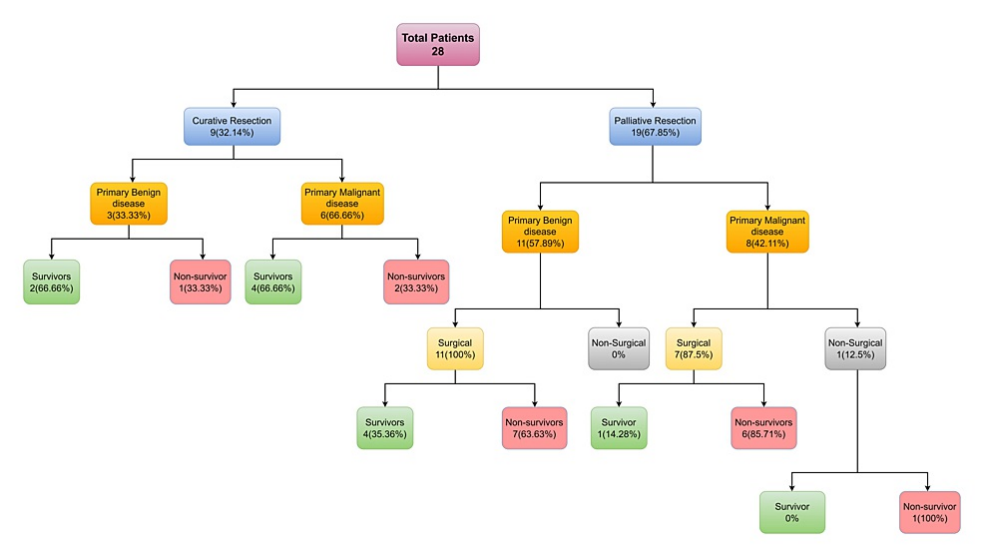
Survival of patients following surgical treatment

A total of nine patients (32.14%) underwent curative resection in the form of total gastrectomy, and enbloc resection of the anastomotic recurrence (Figure 1). Metastatic features such as peritoneal dissemination (n = 8), liver metastasis (n = 6), uterine metastasis (n = 7), and invasion of adjacent organs such as patients with pancreas, jejunum, transverse colon (n = 4), led to 18 (64.28%) patients undergoing non-curative surgery such as bypass surgery (jejunojejunostomy) (n = 4) or feeding jejunostomy (n = 14). Diagnostic laparoscopy was performed in all patients prior to laparotomy. Subsequently, metastatic disease (omentum, peritoneum) was detected in five patients, which was not detected by preoperative evaluation. One patient presented with obstructive jaundice in which a palliative biliary stent was placed.

The Borrmann classification was used for macroscopic examination of specimens. Majority of specimens had type 1, polypoid tumor (75%), with other types such as type 2 (an ulcerated tumor with sharply demarcated margin), type 3 (ulcerated tumor without a demarcated margin and infiltrating to surrounding gastric wall), type 4 (diffuse infiltrating tumor), and type 5 (unclassified) seen in 17.85%, 3.57%, 0%, and 3.57% respectively. The stages of the disease after GSC surgery were divided into stages I, II, III, and IV, which were 0%, 10.71%, 14.28%, and 75%, respectively. Histological differentiation was poorly differentiated adenocarcinoma in 10 (35.71%) patients, moderately differentiated in nine (32.14%), and well-differentiated in one (3.57%). Eight patients were diagnosed with the disseminated disease by analyzing the ascites of malignant cells and examining peritoneal or omental metastases.

All patients were treated with chemotherapy (palliative or adjunctive) which was decided by the multidisciplinary team. At follow-up, a total of 17 patients (60.71%; malignant: 9, benign: 8; p: 0.699) died within six months of GSC surgery. Other patients (39.28%) are doing well. Sixty-six percent of patients who underwent curative resection were successful with follow-up. The survival rate of patients in the curative resection group (n=6; 66.66%) was significantly higher than that in the palliative resection group (n= 5; 26.31%) (p-0.041) (Table 2).

**Table 2:** Survival comparison between curative resection and palliative resection groups

Mortality	Total	Curative resection	Palliative resection	P-value
Survivor	11 (39.3%)	6 (66.7%)	5 (26.3%)	0.041
Non-survivor	17 (60.7%)	3 (33.3%)	14 (73.7%)	
Total	28 (100%)	9 (100%)	19 (100%)	

Univariate analysis of clinicopathological factors related to short-term outcomes after GSC surgery (Table 3) and factors affecting the time interval between GSC occurrences (Table 4) was not significant in this study.

**Table 3:** Univariate analysis of factors associated with mortality in the study population GSC: gastric stump carcinoma; TV/GJ: truncal vagotomy/gastrojejunostomy; JJ/FJ: jenunojejunostomy/feeding jejunostomy; WDAC: well-differentiated adenocarcinoma; MDAC: moderately differentiated adenocarcinoma; PDAC: poorly differentiated adenocarcinoma

Factor	N (%)	Odds ratio	95% CI	P-value
Age (years)		1.044	0.973 – 1.120	0.233
Gender				
Female	6 (35.3%)	-	-	-
Male	11 (64.7%)	0.655	0.139 – 3.079	0.592
Primary procedure				
TV/GJ	9 (52.9%)	-	-	-
Subtotal gastrectomy	8 (47.1%)	1.067	0.233 – 4.885	0.934
Adjuvant therapy (after primary surgery)				
Not received	9 (54.5%)	-	-	-
Received full course	7 (41.2%)	1.167	0.234 – 5.808	0.851
Defaulter	1 (5.9%)	0.667	0.035 – 12.84	0.788
Time interval for the occurrence of GSC (years)		0.996	0.931 – 1.065	0.900
5 -10 years	9 (52.9%)	-	-	-
10-20 years	3 (17.6%)	0.833	0.102 – 6.783	0.865
>20 years	5 (29.4%)	0.694	0.126 – 3.839	0.676
Site of recurrence				
Non-anastomotic	6 (35.3%)	-	-	-
Anastomotic	11 (64.7%)	0.688	0.131 – 3.610	0.658
Borrmann type (GSC)				
1	12 (70.6%)			
2	3 (17.6%)	0.964	0.134 – 6.95	0.971
3	1 (5.9%)	-	-	-
5	1 (5.9%)	-	-	-
Type of procedure performed for GSC				
Feeding jejunostomy	11 (64.70%)	-	-	-
JJ/FJ	3 (17.64%)	0.818	0.061 – 10.99	0.880
Total gastrectomy	1 (5.9%)	0.136	0.009 – 2.068	0.151
Resection of recurrence	2 (11.8%)	0.273	0.026 – 2.829	0.276
Pathological stage (after surgery for GSC)				
Stage 2	1 (5.9%)	-	-	-
Stage 4	16 (94.1%)	6.400	0.474 – 86.343	0.162
Pathological differentiation after surgery for GSC				
WDAC	1 (5.9%)	-	-	-
MDAC	6 (35.29%)	0	-	1.000
PDAC	10 (58.82%)	0	-	1.000
Metastatic site				
Liver	6 (35.305)	5.45	0.556 – 53.523	0.145
Omentum	7 (41.2%)	3.15	0.515 – 19.27	0.214
Peritoneum	8 (47.1%)	4.00	0.659 – 24.29	0.132
Adjacent organs (pancreas, jejunum, transverse colon)	4 (23.5%)	3.077	0.296 – 31.98	0.347

**Table 4:** Clinicopathological factors affecting the time interval for the occurrence of GSC GSC: gastric stump carcinoma; TV/GJ: truncal vagotomy/gastrojejunostomy; WDAC: well-differentiated adenocarcinoma; MDAC: moderately differentiated adenocarcinoma; PDAC: poorly differentiated adenocarcinoma

Primary procedure	Total	Interval (in years)				P-value
		5	6	7	8	
TV/GJ	1 (7.1%)	0 (0%)	1 (20%)	0 (0%)	0 (0%)	0.585
Subtotal gastrectomy	13 (92.9%)	5 (100%)	4 (80%)	3 (100%)	1 (100%)	
Grade of differentiation (after primary surgery)	Total	Interval (in years)				P-value
		5	6	7	8	
WDAC	1 (7.1%)	0 (0%)	1 (20%)	0 (0%)	0 (0%)	0.828
MDAC	5 (35.7%)	2 (40%)	2 (40%)	1 (33.3%)	0 (0%)	
PDAC	8 (57.1%)	3 (60%)	2 (40%)	2 (66.7%)	1 (100%)	
Total	14 (100%)	5 (100%)	5 (100%)	3 (100%)	1 (100%)	
Pathological stage (after primary surgery)	Total	Interval (in years)				P-value
		5	6	7	8	
Stage 1	1 (7.1%)	0 (0%)	0 (0%)	1 (33.3%)	0 (0%)	0.520
Stage 2	4 (28.6%)	1 (20%)	2 (40%)	1 (33.3%)	0 (0%)	
Stage 3	9 (64.3%)	4 (80%)	3 (60%)	1 (33.3%)	1 (100%)	
Total	14 (100%)	5 (100%)	5 (100%)	3 (100%)	1 (100%)	
Adjuvant therapy (after primary surgery)	Total	Interval (in years)				P-value
		5	6	7	8	
Received full course	11(78.6%)	5 (100%)	3 (60%)	2 (66.7%)	1 (100%)	0.655
Defaulter	2(14.3%)	0 (0%)	1 (20%)	1 (33.3%)	0 (0%)	
Not received	1 (7.1%)	0 (0%)	1 (20%)	0 (0%)	0 (0%)	
Total	14 (100%)	5 (100%)	5 (100%)	3 (100%)	1 (100%)	

## Discussion

Although gastric cancer has decreased in recent decades, the prevalence of GSC has not yet decreased due to the long latency period after primary gastric surgery. Therefore, GSC will continue to be a major medical issue for decades to come. Male patients are considered to be at greater risk of being affected by GSC compared to females. This may be related to the high prevalence of primary gastric cancer in men, and also because estrogen production prevents gastric cancer in women. It was reported that the ratio of males to females having GSC after gastric cancer is 3.1: 1 [10]. In contrast, our study showed a male-female ratio of 0.75:1 in GSC cases after gastric cancer surgery.

Lymph node metastasis plays an important role in gastric cancer. It is also an important prognostic factor for GSC. Primary lymphatic drainage usually flows along the gastric cardia region, the left gastric artery, and the splenic artery. Therefore, radical lymph node dissection is still necessary, as recommended by the Japan Gastric Cancer Association [8]. In addition, the outflow route of lymph node metastasis is along the anastomotic site of the jejunum [11]. In one cohort, 16 of 94 patients (17.0%) had mesenteric lymph node metastases after Billroth II anastomosis [12]. Therefore, resection of the jejunal-mesentery near the anastomotic site is still recommended for patients undergoing Billroth II anastomosis.

Our study shows that GSC tends to originate from the anastomotic site regardless of the primary disease. Lee et al. [13] and Ojima et al. [14] reported that tumor location did not significantly affect survival. In contrast, Firat et al. [15] stated that tumor location at the anastomotic site is possibly a good prognostic factor. On the other hand, Namikawa et al. [16] found that patients with tumors in the anastomotic site had a poor prognosis. Therefore, the importance of tumor location for survival is still controversial.

The recurrence pattern of GSC was similar to that previously reported for primary gastric cancer. The results of the available literature on primary gastric cancer recurrence patterns show that peritoneal recurrence is the most common (43-45.9%) and lymph node and local recurrence is the second most common (34%) [16,17]. GSC showed the same trend in this study, with no difference between groups of patients with benign or malignant disease.

Most patients with GSC present at an advanced stage, which results in a low curative resection rate (38-40%) and a poor prognosis [18,19]. In this study, the curative resection rate is 32.14%.

The interval between the index and second surgery depended on the underlying disorder of patients who underwent GSC gastric stump resection. Bile and pancreatic reflux in patients with benign disease have been implicated in the etiology of GSC [20]. Therefore, the onset of GSC can take a long time. On the other hand, in patients with malignant disease, GSC is considered to be metachronous because gastric cancer is generally believed to take 10-20 years to become visible and recognizable [21]. Interestingly, despite these different etiologies that lead to different time intervals in GSC, the oncological features were similar with respect to the prognosis of patients with benign and malignant diseases.

Compared to patients whose GSC was previously associated with gastric ulcer disease, GSC patients with a history of gastric cancer had a relatively good prognosis. Ahn et al. reported that the difference in overall survival between patients who previously had benign disease and those who had a malignant tumor was not significant [22]. Hu et al. stated that patients with GSC with a history of benign disease had a better prognosis than their peers with a history of gastric cancer [23]. In contrast, our study showed that patients with GSC after a history of gastric cancer had a relatively better prognosis than after a history of benign gastric ulcer. GSC patients with a history of gastric cancer were regularly examined at an outpatient clinic and underwent upper gastrointestinal endoscopy, abdominal ultrasonography, and abdominal CT to detect recurrent tumors of the remnant stomach at an early stage. Patients with a history of benign gastric ulcers have a longer incubation period for GSC after gastrectomy. Due to the long incubation period, these patients were not regularly followed up at our clinic. As a result, their GSC was not detected in the early stages. They were diagnosed at an advanced stage. Therefore, the prognosis for patients with GSC associated with a gastric ulcer is relatively worse than their stump recurrence associated with gastric cancer. Patients who have undergone gastrectomy for gastrointestinal ulcers require regular endoscopy of the upper gastrointestinal tract.

Surgical resection of GSC is the only curative option. It is technically difficult due to previous surgery, but possible if diagnosed early in a well-performing patient. Short-term outcomes such as hospital stay, 30-day mortality, and long-term outcomes such as disease-free survival and overall survival were similar between primary gastric cancer and GSC patient groups when curative resection of GSC is possible [24].

Nowadays, patients, especially those who have overcome malignancy, are more concerned about their health. Needless to say, these patients adhere more closely to postoperative follow-up. This will prevent the recurrence of stomach cancer and increase the five-year survival rate. In addition, gastric cancer treatment experience and techniques (especially surgical procedures) are more mature and advanced than in the past. There are few chemotherapeutic agents (S-1, cisplatin, 5FU) for the treatment of GSC, but surgery remained the most effective treatment as long as surgery was performed on properly selected patients [25]. 

Limitations

Our study is a retrospective analysis. Not all GSC patients underwent initial surgery at our institution, so detailed information about the initial surgery for benign diseases was lacking and it was difficult to collect all the information about the initial surgery.

## Conclusions

The appearance of GSC occurs sooner in patients with primary malignancy than in patients with a primary benign disease. Therefore, close follow-up is necessary for at least 10 years after the first gastrectomy, as half of the patients expected to develop GSC will develop it within that period. Curative resection is an independent prognostic factor for survival in patients with GSC. Though the overall survival of patients with GSC is poor, curative resection can improve their prognosis in selected patients, such as those with early-stage disease and good performance status.
